# Recent Advances in Neuro-Psychiatry

**Published:** 1903-12

**Authors:** 


A Monthly Review of Medicine and Surgery.

*EDITOR:*

WILLIAM WARREN POTTER, M. D.

All communications, whether of a literary or business nature, books for review and exchanges should be addressed to the editor: 284 FRANKLIN STREET, BUFFALO, N. Y.

VOL. XLIII.—LIX. DECEMBER, 1903. No. 5.

### Recent Advances in Neuro-psychiatry.

TWO very important neuro-psychiatral publications have recently appeared in medical literature, the one emanating from the biological laboratory of the Sheppard and Enoch Pratt Hospital, at Baltimore, the other from the laboratory of the neurological clinic at Halle, Germany, under the directorship of Professor Eduard Hitzig. Both deal with the intricate mechanism of brain function and brain action, the latter, however, dealing with the physiological, the former with the pathological side of the question. The Sheppard-Pratt publication, under the directorship of Dr. Edward N. Brush, is composed of a series of articles by members of the staff, on subjects relating to pathological changes in the brain and the symptoms produced by such lesions, while Professor Hitzig's monograph consists of an elaborate study on the relation of the cortex and subcortical ganglia to vision.

About 34 years ago, Professor Hitzig first gave to the world his marvelous discovery of the motor center in the brain. In connection with Professor Fritsch, by means of galvanic stimulation of the dog's cortex, they confirmed the conclusions of Hughlings Jackson, of London, and established beyond dispute the existence of motor centers in the brain. Following these men came Ferrier, Munk, Schiff, Horsley and Beevor, Goltz, Bastian, Bianchi, Sherrington, Grünbaum and others, differing somewhat in minor details, but all aiding to establish the original premise of Hitzig, that certain parts of the cortex are intimately associated with motor phenomena, while the remaining parts have no such direct relation.

Curiously enough in the Sheppard-Pratt report appears an excellent article on the motor cortex by Clarence B. Farrar, a clinical assistant of the hospital, who has reviewed the work done since

1870 from the speculative clinico-pathologic, physiologic, anatomic and embryologic viewpoints, and in his very able résumé finds naught to mar Hitzig's contention of 34 years standing, except that the confines of motor cortex-excitability have gradually become narrowed, so as to include only the middle region of the hemispheres, intermediate between the general and special sensory areas on the one side, and the specific association or psychic center on the other.

Professor Hitzig has grown to be an old man, so old that one can not help but be moved by his pathos and his regret at leaving the field of cerebral localisation. He says in concluding his work, comprising over 400 pages, for the most part empirical, that "my investigations regarding the brain are now at an end. My eyesight has almost entirely disappeared and I am suffering with writer's cramp, so that I cannot continue my devoted work any longer. What an example for the younger generation of investigators, what an inspiration, what a figure to look up to! The passing of Professor Hitzig, however, does not mean the decadence and the slumbering of cerebral investigation. The work so eagerly begun by him is being continued throughout the world, and with much energy and perseverance in the United States.

Dr. Brush's report is but a single example of what other institutions are doing along the line. The pathological laboratories of the state institutions are doing work comparing favorably with that done in European laboratories, and the high character and deep scientific spirit is universally acknowledged.

The report of the Sheppard-Pratt Hospital may well be taken as an example, and a closer examination of its contents will reveal the scope and intent of its author.

The director of the laboratory, Stewart Paton, contributes an able article on studies in the manic depressive insanity. In addition to the suggestions made by the author that more extended observations should be made: (1) regarding the variations of the blood pressure, and (2) regarding the changes in the urine during the period of excitement or depression, he might have added also that the functions of the liver should be especially noted for cholemic influence. Clarence B. Farrar contributes an interesting article on the typhoid psychoses. After narrating four cases and discussing briefly the current teaching concerning the effects of the typhoid process on mental functions, the author finally concludes that there, neither in its clinical or anatomical picture, is the typhoid psychosis distinctive.

The next following brief article by William Rush Dunston, Jr., on some points in the diagnosis of dementia precox, is devoted mainly to the pointing out of certain symptoms which the author found helpful in differentiating dementia precox from neurasthenia and the recovery psychoses. The most important of these is the slow psychical reaction or psycho-motor retardation, having been found present in every case observed by the author. A case of Huntington's chorea with autopsy is the next paper contributed by Glanville Y. Rusk. The author gives the clinical history of a case of this rare disease, with a minutely described microscopical examination of the brain.

Dr. Clarence B. Farrar's article on the motor cortex has been alluded to. Another carefully examined case is that on acute paresis, by Dr. Stewart Paton and Dr. G. Y. Rusk. The microscopical findings revealed nothing that was essentially characteristic of the disease; the character of the changes in the various organs as well as in the central nervous system suggests a general intoxication. Two handsomely engraved plates accompany this article, showing large and small pyramidal cells from the cerebral cortex, bloodvessels, and neuroglia cells. The concluding article is by Dr. William Rush Dunton, Jr., on a case of dementia precox, with autopsy. The microscopical examination showed the greatest amount of cell changes in the first frontal convolution, with slight increase of neuroglia nuclei, phagocytosis and considerable cell disintegration. Four handsome plates accompany this article.

There must be considerable satisfaction to Dr. Brush for being the sponsor of a report from his institution, so highly scientific and thoroughly mastered as this, which has been so inadequately outlined in this review. He has set a good model for similar work by other institutions.

---

At the recent meeting of the American Public Health Association held at Washington, the committee on vital statistics reported that effective coöperation had been instituted between that association, the Conference of State Boards of Health, the American Medical Association, the United States Census Bureau and the United States Public Health and Marine Hospital Service for the improvement of the vital statistics of this country. Among the objects sought are the extension of adequate methods of registration, the use of uniform and comparable tables and rates in bulletins and reports, and the improvement of the international classification of causes of death. A pamphlet on "Statistical Treatment of Causes of Death" has been issued by the United States Census Bureau, requests for which should be addressed to Mr. W. A. King, chief statistician for vital statistics, Census Bureau. It has special reference to the difficulties encountered in compiling deaths returned from several causes, and asks for the coöperation of the profession in framing a thoroughly satisfactory method of procedure in such cases.

In the edition of the JOURNAL for July, 1903, we made somewhat extended editorial reference to the Manual of International Classification of Causes of Death, issued by the census office and we are glad to have an opportunity to refer to the subject again in the light of the action of the American Public Health Association. A uniformity of practice upon this important question cannot too soon prevail. Every educated physician should give it earnest thought.

---

WE PRINT elsewhere in this issue an article from the pen of Dr. D. W. Cathell, of Baltimore, dealing interestingly and in considerable detail with the action of the American Medical Association at New Orleans, last May, in abolishing the code of ethics that so long has hung over the medical profession like a pall, and from which it was so happily delivered through the splendid diplomacy of Dr. Charles A. L. Reed, of Cincinnati. Lest it be forgotten, perhaps it may be well to repeat briefly the facts that led up to the change. When the committee on ethics appointed at Saratoga, presented its report at New Orleans through its chairman, Dr. Harris of New York, there was nothing to indicate that the house would not reënact a code of ethics even more offensive than the one ablated at Saint Paul in 1901. But Dr. Reed obtained the floor immediately and moved a substitute, which gave him the aggressive side of the question in leading a determined opposition to the code and which at the same time put the other side on the defensive. The whole question was then referred to a committee of one from each state, which should include the Saratoga committee and Dr. Reed. The latter found a strong coadjutor in Dr. William H. Welch, of Baltimore, and the enlarged committee had not long been in session before it was discovered that victory was assured for Dr. Reed and his inclining.

We hope Dr. Cathell's article will be read carefully by every friend of the old code. It is a thoughtfully prepared analysis by a man who until recently was an ardent champion of that remarkable instrument which, though well enough in its day, for the past twenty years has represented a bygone age.

---

THE following paragraphs recently clipped from the *New York Tribune,* show the intelligent side of newspaper medicine:

Once more the cancer parasite has been found, this time by a German, Dr. Schmidt. He has also manufactured a serum for that germ, and hopes to secure remedial effects thereby. Similar discoveries have been reported so often that there is a decent excuse for receiving the latest story with caution. In matters of this kind it is better to hope too little than too much, so long as corroboration is lacking.

The admirable work done by St. Luke's Hospital is to be extended by the erection of a new pavilion, the gift of Mrs. Margaret J. Plant. The original plans provided for the construction of ten such pavilions, five of which are already in use. This excellent institution has an ideal site on Morningside Heights, and its beneficent operations have been of inestimable usefulness.

An obelisk of unpolished gray granite has been placed over Virchow's grave, in the old Matthäi graveyard, Berlin. It bears on one side a black marble tablet, on which are inscribed "Rudolph Virchow" and the date of his birth and death. A statue of Virchow will also be erected near the place where his scientific work was conducted.

Caution should be exercised in accepting all stories about new remedies for old maladies, but there is an exceptional reason for questioning their efficacy in cases of tuberculosis. It has been learned from post-mortem examinations that an extraordinarily large number of people are affected with that disease without knowing it, and completely recover without having a suspicion. It may be the unassisted ability of thousands to shake off the enemy, rather than the virtues of the alleged specific, which explains the marvelous "consumption cures" so frequently reported nowadays.

